# Standing out from the crowd: Both cue numerosity and social information affect attention in multi-agent contexts

**DOI:** 10.1177/17470218211013028

**Published:** 2021-04-29

**Authors:** Francesca Capozzi, Andrew P Bayliss, Jelena Ristic

**Affiliations:** 1 Department of Psychology, McGill University, Montreal, QC, Canada; 2 School of Psychology, University of East Anglia, Norwich, UK

**Keywords:** Gaze following, group interactions, learning effects, social attention

## Abstract

Groups of people offer abundant opportunities for social interactions. We used a two-phase task to investigate how social cue numerosity and social information about an individual affected attentional allocation in such multi-agent settings. The learning phase was a standard gaze-cuing procedure in which a stimulus face could be either uninformative or informative about the upcoming target. The test phase was a group-cuing procedure in which the stimulus faces from the learning phase were presented in groups of three. The target could either be cued by the group minority (i.e., one face) or majority (i.e., two faces) or by uninformative or informative stimulus faces. Results showed an effect of cue numerosity, whereby responses were faster to targets cued by the group majority than the group minority. However, responses to targets cued by informative identities included in the group minority were as fast as responses to targets cued by the group majority. Thus, previously learned social information about an individual was able to offset the general enhancement of cue numerosity, revealing a complex interplay between cue numerosity and social information in guiding attention in multi-agent settings.

Every day, whether at a park or a supermarket, we encounter multiple people who offer abundant opportunities for social interactions. Humans navigate these complex multi-agent situations effortlessly despite the burden that the amount of social information often exerts on our socio-cognitive capacities. The attentional system, which allows us to select and respond to the most relevant social cues, is likely one of the key cognitive mechanisms that support such complex social behaviour (Capozzi & Ristic, 2018; Meerhoff et al., 2018). Here we investigated how social cue numerosity and social information about an individual affect attentional allocation in multi-agent social settings.

Gaze following, defined as the spontaneous orienting of attention towards others’ gaze direction (Capozzi & Ristic, 2020), is a basic social attentional behaviour that can occur both overtly (i.e., attention is located by executing eye movements) and covertly (i.e., attention is located without executing eye movements; Dalmaso et al., 2020). Covertly, gaze following is typically experimentally investigated using a computerised cuing procedure, in which a stimulus face presented on a computer screen shifts their gaze towards or away from an upcoming response target. Gaze following is demonstrated by faster responses for gazed-at relative to not gazed-at targets (Frischen et al., 2007). In multi-agent settings, multiple people often look in different directions, which may overwhelm the attentional system if all gaze directions were followed. Research on gaze following in these scenarios shows that gaze direction of the group majority is often prioritised relative to the gaze direction of the group minority indicating that cue numerosity is an important factor in guiding attentional responses to inconsistent gaze directions (Capozzi et al., 2018, 2021; Sun et al., 2017).

Research has also shown that social information about an individual, such as learning that they may be competent or reliable (Capozzi et al., 2016), is also an important factor in gaze following. For example, social information like social competence (Capozzi et al., 2016), reliability (Dalmaso et al., 2015), and communicative salience (Carlson & Aday, 2018) have all been found to modulate attentional responses to gaze such that the gaze of individuals perceived as more competent, reliable, or communicative elicits greater magnitudes of gaze following relative to gaze of individuals perceived as carrying lower levels of social information (Capozzi & Ristic, 2018). Often, learning paradigms that manipulate social information about experimental identities are used to convey such information in laboratory settings (e.g., Bayliss & Tipper, 2006; Capozzi et al., 2016; Dalmaso et al., 2015; Joyce et al., 2016; Rogers et al., 2014) with results showing a complex relationship between social information learning and later gaze-cuing responses. For example, while some studies have found that learning about individuals’ social competence (Capozzi et al., 2016) increases subsequent gaze-following behaviour, other studies have found that learning about social reliability decreases those behaviours (Dalmaso et al., 2015; Joyce et al., 2016). Thus, while both cue numerosity and social information have been found to affect gaze following, an open question remains how and whether these two variables jointly affect gaze following in multi-agent settings. This question is at the centre of the present investigation.

To study how cue numerosity and social information affect gaze following, we used a two-phase task with a learning phase and a test phase (see, for example, Capozzi et al., 2016). In the learning phase, we exposed participants to a standard gaze-cuing procedure in which a stimulus face could be either uninformative or informative about the upcoming target, such that participants could learn about the social reliability of the gazing identities in correctly cuing the target’s location. Although social information can be manipulated in multiple ways, including categorization in terms of personal factors such as age, gender, or social status (Capozzi et al., 2016; Ciardo et al., 2014; Dalmaso et al., 2012; Jones et al., 2010), here we used a general procedure for social information learning that could be inferred from gaze behaviour independently from other stable social characteristics. Similar procedures have been previously found effective in instantiating social learning by manipulating social information with respect to the perceived reliability of the stimulus identities (Capozzi et al., 2016; Dalmaso et al., 2015; Rogers et al., 2014) while also minimising interaction effects between participants and stimuli due, for example, to perceived similarity (e.g., Dalmaso et al., 2020; see also Ciardo et al., 2014).

Then, to investigate the links between cue numerosity and social information, in the test phase of the present study, participants re-encountered those same face identities which were now presented in groups of three. To assess the effects of cue numerosity, we manipulated the group-ratio that cued the target *and* examined responses to targets appearing at the location cued by the group minority (i.e., one face) or group majority (i.e., two faces). To additionally assess the effects of learned social information, we also examined whether these responses were modulated by whether the socially informative identity was part of the group’s minority or group’s majority. In this way, we were able to investigate the interactions between the quantity and the quality of social information in guiding attention in multi-agent contexts by pitting the attentional effects of the quantity (i.e., the number) of socially uninformative agents against the perceived quality (i.e., social value) of informative agents.

There are two possible outcomes how social information and cue numerosity may relate to influence social orienting. One possibility is that group majority would always outperform group minority, independent of the social information of the individuals composing the group. Alternatively, social information may offset the general enhancement of cue numerosity when the informative identity appears in the groups’ minority suggesting that the perceived quality of social information guides attention in multi-agent settings as the observed quantity of social information.

## Methods

### Participants

Prior to the experiment, we decided to test about 70 participants. We chose this sample size based on a conservative a priori power analysis with dz = .3, α = .05, β = .20 (Faul et al., 2007; see also Capozzi et al., 2018) given mixed results in previous research on learning effects in gaze-cuing procedures (e.g., Capozzi et al., 2016; Dalmaso et al., 2015; Joyce et al., 2016). Seventy-five McGill undergraduate students (66 females, 9 males; mean age = 21.23 years, age range = 18–35), naive to the purpose of the study, with normal or corrected-to-normal vision, participated in the study in exchange for course credits. All procedures were in accordance with the World Medical Association Declaration of Helsinki (2013) and were approved by the University’s research ethics board.

### Apparatus and stimuli

**Figure 1.**
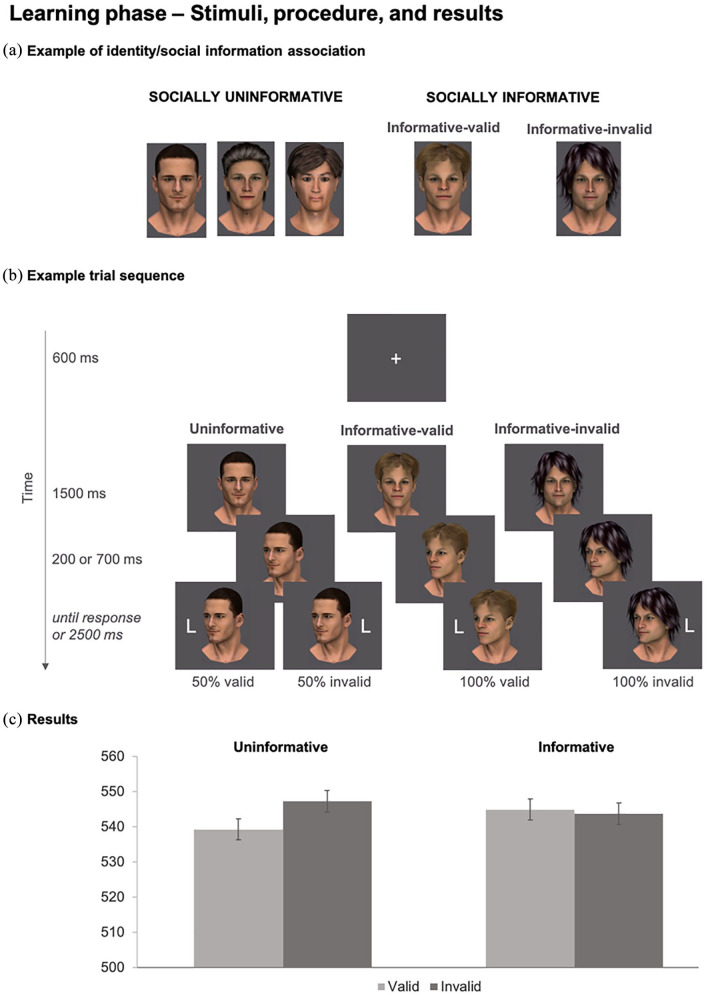
Learning phase. (a) Socially uninformative and socially informative identities. (b) Example trial sequence and social information manipulation. (c) Results showed as a function of social information and cue validity. Drawings are not to scale. Error bars indicate standard error of the mean for within-subject designs (Loftus & Masson, 1994).

**Figure 2.**
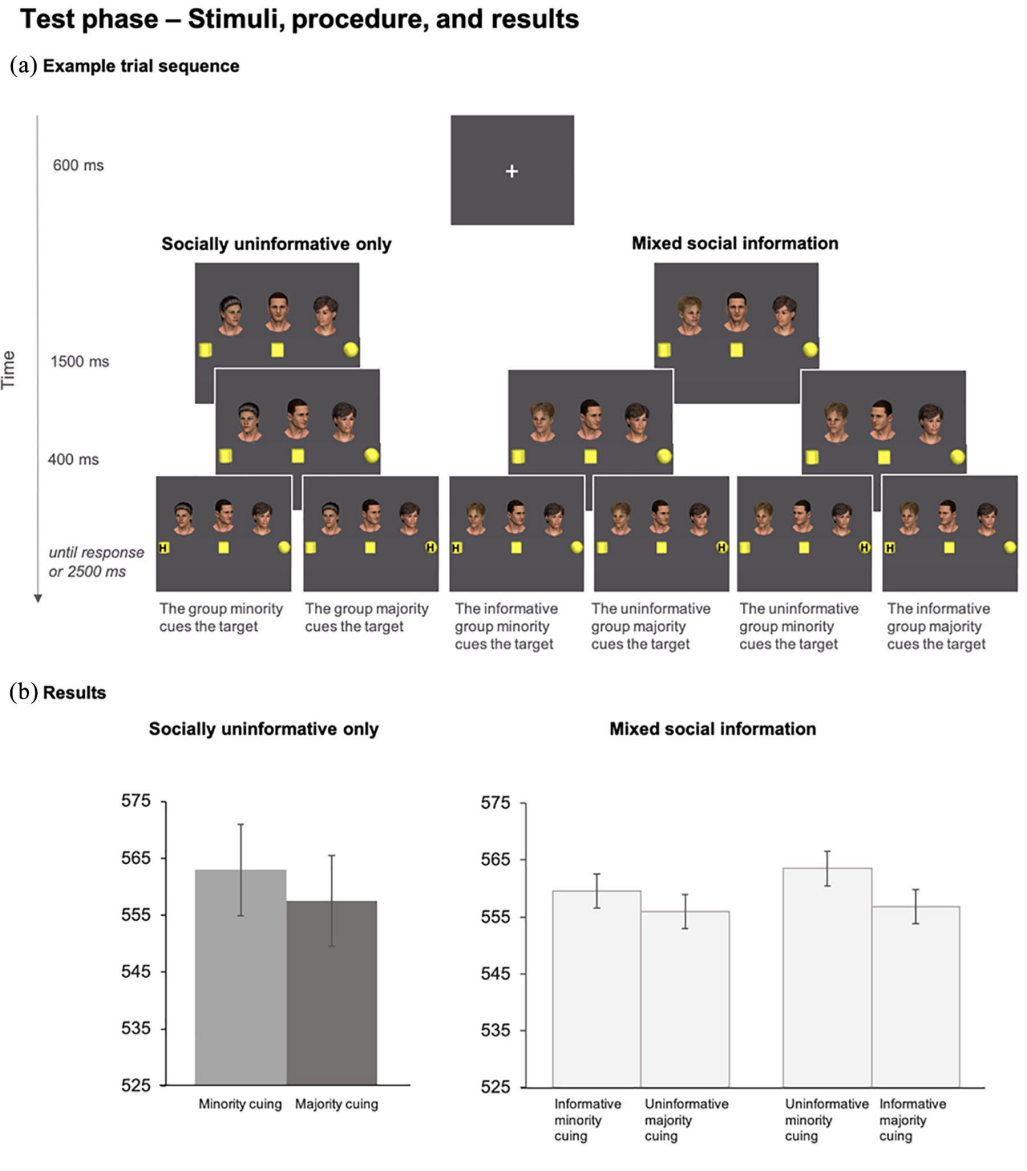
Sample trials using an informative-valid example (a) and results (b) for the test phase. Drawings are not to scale. Error bars indicate standard error of the mean for within-subject designs (Loftus & Masson, 1994).

Stimulus presentation and data collection were controlled by Experiment Builder (SR Research). The stimulus sequence was presented on a 16-inch CRT monitor connected to a personal computer at an approximate viewing distance of 60 cm. Stimuli are shown in Figure 1. Following previous research (Capozzi et al., 2018, 2021), stimuli included colour images of five male faces created using Smith Micro’s Poser 9 software. The images were set against a grey background and varied in size from 4.20° to 4.55° in width and 6.77° to 7.63° in height. The capital letters T and L (1.43° × 1.90°) served as response targets. The test phase (depicted in Figure 2) additionally included three yellow coloured placeholder objects—a cube, a cylinder, and a sphere (varying in size from 2.00° to 2.39° in width and height)—which were used to facilitate inference of line of sight (see also Capozzi et al., 2016, 2018, 2021). The capital letters H and N (1.43° × 1.90°) served as response targets.

### Design

The study was a within-subject design, with a Learning phase and a Test phase completed by all participants in this order.

#### Learning

The learning phase consisted of a gaze-cuing task, in which Cue validity, Social information, and Cue-target interval were manipulated. *Cue validity* refers to the combination of gaze direction and target location. Valid trials denote the trials in which the target appeared at the same location as indicated by the face’s gaze direction. Invalid trials denote the trials in which the target appeared in the opposite location than indicated by the face’s gaze direction. As shown in Figure 1a, *Social information* manipulation involved imbuing each face identity with information about their reliability in cuing the target. In the uninformative trials, as depicted in Figure 1b, gaze cues from three face identities provided no reliable information about the location of the target such that the target occurred at the gazed-at location in 50% of trials and at the not gazed-at location in the remaining 50% of the trials. In the informative trials, the remaining two face identities’ gaze cues provided information about the target location. Specifically, one face identity (informative-valid) consistently gazed at the correct target location whereas the other face identity (informative-invalid) consistently gazed away from the correct target location. Finally, *Cue-target interval* manipulated the time between the onset of the cue display and the onset of the target between 200 (short interval) and 700 ms (long interval). This was included as a typical cuing task parameter (Frischen et al., 2007). All factors were manipulated randomly and equiprobably, with each face identity appearing the same number of times.

#### Test

The test phase consisted of a modified gaze-cuing task in which three stimulus faces from the prior learning phase were simultaneously presented in a group configuration, as shown in Figure 2a. Here, depending on the type of trial, cue numerosity and social information were individually or jointly manipulated. In the “Socially uninformative only” trials (92/288), the group consisted of the three socially uninformative identities which did not provide any reliable social information at Learning. To manipulate cue numerosity, the factor *Group-ratio cuing* manipulated whether the target was cued by the group minority (i.e., one face) or majority (i.e., two faces). These trials were included to validate any effect of cue numerosity independently of social information.

In the “Mixed social information trials” (192/288), identities imbued with social information in the learning phase were now included in the group. In these trials, along with two uninformative faces, one of the faces was an informative identity that was either informative-valid or informative-invalid at learning. Here cue numerosity and social information were manipulated jointly such that the target could still be cued by the group minority or majority, but it could be now cued by the informative identity included either as part of the group minority (i.e., minority informative) or as a part of the group majority (i.e., majority informative; Figure 2a). That is, the group-ratio was qualified by social information such that one socially informative individual (informative minority) could be presented along with two socially uninformative individuals (uninformative majority) or, vice versa, one socially uninformative individual (uninformative minority) could be presented along with an informative majority composed by one informative and one uninformative individual. Overall then, the factor *Information type* (informative-valid vs. informative-invalid) manipulated whether the socially informative faces gave informative-valid or informative-invalid information at learning, the factor *Group-ratio social information* (minority informative/majority uninformative vs. majority informative/minority uninformative) manipulated how social information was distributed across the group, and the factor *Group-ratio cuing* (minority cuing the target vs. majority cuing the target) manipulated whether the target was cued by the group’s majority or minority.

### Procedure

#### Learning phase

The learning phase procedure is illustrated in Figure 1b. Each trial started with a presentation of a fixation cross (600 ms) followed by the image of one of the face identities displaying straight-ahead gaze for 1,500 ms. Then, the face image was shown with their head turned towards the left or the right for 200 or 700 ms (50% trials each). Finally, a response target (a capital letter T or L) appeared on the left or right of fixation, and participants were instructed to press one of two adjacent keyboard keys (V and B) marked in yellow and blue depending on target identity. Target identity—key assignment was counterbalanced across participants. Participants were told to ignore the gaze shift and identify the target as quickly and accurately as possible. A tone sounded upon missed erroneous responses. Intertrial interval was 600 ms. After 16 practice trials, in which only the targets appeared, the learning phase proceeded over 200 trials split over four blocks and took approximately 15 min. Each face was presented for 40 trials for an overall equal number of valid and invalid trials and short and long cue-target intervals (see also Supplemental Material).^1^

#### Test phase

The Test phase procedure is illustrated in Figure 2a. After the presentation of a fixation cross (600 ms), a display showing the three faces turned towards the central placeholder was shown. After 1,500 ms, one face shifted their gaze towards one lateral object, while the other two faces shifted their gaze towards the other lateral object. After 400 ms, a target (a capital letter H or N), requiring an identification response, was presented on either the left or right object. Participants were instructed to identify the target quickly and accurately by pressing one of two adjacent keyboard keys (V and B) marked in yellow and blue, with target identity—key assignment counterbalanced. The faces and the target remained visible until response or until 2,500 ms had elapsed. A tone sounded upon missed or erroneous response. Intertrial interval was 600 ms.

Participants were instructed to ignore the face cues, and to maintain central fixation. After 16 practice trials, in which only response targets appeared, the experiment proceeded over 288 experimental trials and 36 additional catch trials in which no target appeared. The total 324 trials were divided into three blocks and took approximately 25 min to complete.

## Results

### Learning

We examined response accuracy and mean RT using repeated measures analyses of variance (ANOVAs), run as a function of *Cue validity* (valid, invalid), *Social information* (uninformative, informative), and *Cue-target interval* (short, long). Participants performed the task well, with 96% overall accuracy, and no evidence of speed-accuracy trade-off based on *Cue validity*, *F*(1, 74) = 5.538, *p* = .021, $\eta_{p}^{2}$ = .070, whereby responses to valid targets (*M* = 95.46, 95% CI [94.62, 96.29]) were overall more accurate than responses to invalid targets (*M* = 94.56, 95% CI [93.48, 95.66]), and no additional differential effects across experimental conditions (*F*s < 2.045, .157 < *p*s < .999).

RT analyses were conducted on correct trials and additionally excluded any anticipatory and timed-out responses (i.e., responses faster than 200 ms and slower than 1,200 ms, 1.28% of trials). The analysis revealed a main effect of *Cue-target interval*, *F*(1, 74) = 133.706, *p* < .001, $\eta_{p}^{2}$ = .644, indicating a typical foreperiod effect with faster overall responses at long (*M* = 527, 95% CI [597, 546]) relative to short (*M* = 561, 95% CI [540, 582]) intervals. The analysis also revealed a two-way interaction between *Cue validity* and *Social information*, *F*(1, 74) = 4.331, *p* = .041, $\eta_{p}^{2}$ = .055, plotted in Figure 1c. No other effects were significant (*F*s < 2.848, .096 < *p*s < .782).

We followed up on the interaction between *Cue validity* and *Social information* with post hoc pairwise *t* tests, two tailed. These tests showed that the uninformative faces elicited the typical response advantage for valid (*M* = 539, 95% CI [520, 559]) relative to invalid (*M* = 547, 95% CI [527, 567]) trials, *t*(74) = 2.865, *p* = .005, dz = .331, whereas the informative faces did not, *t*(74) = .371, *p* = .711, dz = .074, informative-valid: (*M* = 545, 95% CI [524, 566]); informative-invalid (*M* = 544, 95% CI, 523, 563]). This result appears to reflect an increase in RT for the informative-valid faces relative to the uninformative faces in valid trials, *t*(74) = 1.982, *p* = .051, dz = .209 (see Figure 1c) and was additionally supported by a Bayes Factor (BF = 0.13) modelled on the typical gaze-cuing effect found for the uninformative identities (normal distribution with *M*_difference_ = −8.03 and *SD*_difference_ = 24.26, two tailed). As BFs above 3 are conventionally interpreted as providing substantial support for the alternative hypothesis and those below 0.33 as substantial support for the null hypothesis (Dienes, 2014), these additional analyses support the present finding.

Overall, the results from the learning phase showed that when participants encountered socially uninformative identities, they followed their gaze in a typical manner but did not do so when they encountered socially informative identities. This was mainly due to slow responses to the targets that were cued by the informative-valid identity relative to the targets that were cued by the uninformative identities. This result is partially consistent with past literature that has showed similar validity-learning effects (Joyce et al., 2016) and suggests that the learning phase succeeded in forming an association between the stimulus identities and their expected gaze behaviour.

### Test

As before, RT analyses were conducted on correct trials (94.50% of trials), and additionally excluded anticipatory and timed-out responses (1.81% of trials). Overall, paired two-tailed *t* tests confirmed that irrespective of face identity, when RTs for targets cued by the group minority versus the group majority were compared, faster responses were overall found when the target was cued by the group majority (*M* = 556, 95% CI [539, 574]) relative to the group minority (*M* = 562, 95% CI [543, 580]), *t*(74) = 2.579, *p* = .012, dz = .287.

Due to the nature of the design, a fully factorial ANOVA could not be conducted because the “Socially uninformative only” and “Mixed social information” trials could not be analysed together as the absence of socially informative identities in the “Socially uninformative only” condition excludes the Information type (i.e., informative-valid vs. informative-invalid) and Group-ratio social information factors (minority informative/majority uninformative vs. majority informative/minority uninformative). Thus, separate analyses were necessary to test the interactions between social information and cue numerosity in “Mixed social information” trials.

We first analysed the “Socially uninformative only” trials in which only uninformative face identities appeared. As illustrated in Figure 2b, a paired two-tailed t test comparing the RTs for targets cued by the group minority versus the group majority confirmed an effect of cue numerosity indicating overall faster responses when the target was cued by the group majority (M = 557, 95% CI [540, 575]) relative to when it was cued the group minority (M = 563, 95% CI [545, 581]), t(74) = 2.021, p = .047, dz = .259. Thus, the gaze direction of the group majority elicited stronger attentional responses than gaze direction of the group minority when the group was composed of perceived uninformative individuals.

We next analysed the “Mixed social information” trials in which both uninformative and informative faces appeared. Here we used a repeated measures ANOVA run as a function of the type of informative identity that was present in the group (Information type; informative-valid vs. informative-invalid), of how social information was distributed across group minority or majority (Group-ratio social information; minority informative/majority uninformative vs. majority informative/minority uninformative), and of whether the target was cued by the group minority or majority (Group-ratio cuing; minority vs. majority cuing the target).

The analysis revealed a main effect of Information type, F(1,74) = 6.140, p = .015, $\eta_{p}^{2}$ = .077, whereby responses were overall faster when an informative-invalid identity was in the group (M = 557, 95% CI [538, 575]) relative to an informative-valid identity (M = 561, 95% CI [543, 580]). It also indicated an interaction between Group-ratio cuing and Group-ratio social information, F(1, 74) = 4.211, p = .047, $\eta_{p}^{2}$ = .055, and no other main effect or interactions (Fs < 1.995, .162 > ps > .737).

We followed up on the interaction between Group-ratio cuing and Group-ratio social information using two-tailed pairwise t tests. Confirming an overall effect of numerosity, and as depicted in Figure 2b, these tests showed that responses were faster when the group majority cued the target relative to the uninformative minority (M = 564, 95% CI [545, 582]), both when the group majority included an informative face (M = 557, 95% CI [538, 576]), t(74) = 2.135, p = .036, dz = .261, and when it did not (M = 556, 95% CI [537, 575]), t(74) = 2.624, p = .011, dz = .320. However, the group majority did not reliably outperform the group minority when the group minority included an informative identity (M = 559, 95% CI[540, 579]). This was true both when the group majority included an informative face, t(74) = 1.142, p = .257, dz = .110, and when it did not, t(74) = .829, p = .410, dz = .072. These findings were both supported by Bayes Factors (BF = 0.25 and BF = 0.19, respectively) modelled on the difference between RT to targets cued by the group minority versus majority with uninformative identities (normal distribution with M_difference_ = 5.45 and SD_difference_ = 23.37, two tailed), indicating that responses were reliably similar across targets cued by the informative minority and both the informative and uninformative majority. These results show that the gaze direction of the group minority composed of informative agents elicited similar attentional responses as the group majority.

Thus, overall these data confirm a general enhancement for the group majority but additionally suggest that individual social information is integrated in the attentional processing to partially offset the advantage of numerosity.

## Discussion

Multi-agent contexts offer multiple and often inconsistent social cues (Capozzi & Ristic, 2018). Here, we investigated the interplay between cue numerosity and social information in guiding attentional responses in these complex scenarios. We used a learning procedure to manipulate social information and then tested how this information interacted with cue numerosity in eliciting gaze-following responses in a subsequent group-cuing procedure (e.g., Capozzi et al., 2018). Our results showed a general effect of cue numerosity, whereby the gaze direction of the group majority elicited stronger attentional responses than the gaze direction of the group minority. However, previously learned social information about an individual was able to counteract the general enhancement of cue numerosity when the socially informative identity was included in the group minority. That is, the perceived quality of social information was as effective as the observed quantity of social information in guiding observers’ attention. Together, these results raise at the least three points for discussion.

First, we manipulated social information using a learning procedure that has been previously implemented to manipulate social information in paradigms that investigated gaze following in response to the gaze cues of a single individual (e.g., Joyce et al., 2016; see also Capozzi et al., 2016). The results of our learning phase showed the counter-intuitive finding that response times to targets cued by informative-valid identities (i.e., they always cued the target) were similar to those cued by informative-invalid identities (i.e., they never cued the target). This sort of “counter-cuing” is consistent with previous research that has used similar learning paradigms (Joyce et al., 2016; but see Barbato et al., 2020) and has been suggested to occur because the encoding of the expected gaze behaviour interferes with and slows down the attentional response (see also Morgan et al., 2014). Additional research on the learning effects on gaze following has shown similar findings with, for example, identities that had previously consistently followed participants’ gaze direction later being less effective as gaze cues than identities who never followed the participants’ gaze (Dalmaso et al., 2015; see also Edwards et al., 2015). These results converge to suggest that social learning has a complex relationship with subsequent social attentional responses (e.g., Dalmaso et al., 2015) and perceived value of social information (e.g., Barbato et al., 2020; Dalmaso et al., 2020). Importantly, however, our results show that social learning—despite its complexity—modulates attentional responses in multi-agent social scenarios. These results are consistent with the idea that the relevance of social cues can depend on the behavioural history of the identities producing those cues (Capozzi et al., 2016) and further extend this notion to show that this learned relevance has an important role in guiding attention in multi-agent contexts. Further research will benefit from a deeper exploration of the interplay between gaze behaviour, context, and social information in guiding social processing and attention in complex multi-agent scenarios (see, for example, Becker, 2010; Capozzi et al., 2016, 2018, 2021; Carlson & Aday, 2018; Sun et al., 2020).

Second, and relatedly, our results suggest that learned social information increases the relevance of cues conveyed by group minority independent of the type of social information that the individual conveyed. That is, gaze of socially informative individuals elicited similar attentional responses irrespective of whether they had always (informative-valid) or never (informative-invalid) cued the target at learning. This suggests that social information in general guides attention in complex scenarios and dovetails with previous models of social information processing suggesting that various types of social information valence (e.g., positive vs. negative) similarly attract attention (Wentura et al., 2000) and enhance processing (Lemerise & Arsenio, 2000) depending on the context (e.g., approach vs. avoidance goals). However, our data also show that the presence of informative-invalid identities in the group elicited faster responses than informative-valid identities, potentially suggesting that different identities, and specifically informative-invalid ones facilitated attentional disengagement from the cues better than informative-valid identities. This would be consistent with the notion that negative social information often elicits avoidance responses (Wentura et al., 2000) and future research will benefit from the combined investigation of social information valence and different contextual goals in complex social settings (see, for example, Gallup et al., 2014).

Third, and perhaps most intriguingly, our results show that (independent of its valence) social information about an individual interacts with and partially offsets the effects of cue numerosity. Previous research has emphasised that cue numerosity guides behavioural responses in both human infants (Pun et al., 2016) and primates (Pun et al., 2017) in an effortless and spontaneous manner. Recent work has also shown that group-ratio estimates (e.g., the identification of a group majority vs. minority) dynamically guide human adults’ attentional responses in a variety of contexts (Capozzi et al., 2018; Gallup et al., 2012; Jorjafki et al., 2018; Sun et al., 2017). Thus, our finding of greater magnitudes of gaze-following responses to the gaze direction of the group majority versus the group minority is consistent with such previous research. Critically, however, the relevance of the gaze direction of the group majority was not able to offset the relevance of the gaze direction of the group minority when the minority was composed by a socially informative individual. That is, the gaze direction of a socially informative individual elicited similar gaze-following responses as the group majority, potentially suggesting that both types of information (cue numerosity and social information) were perceived as carrying similar relevance. This finding is consistent with research showing, for example, that observers preferentially follow the gaze direction of an individual displaying fearful facial expressions in multi-agent settings with competing gaze cues (Becker, 2010; Carlson & Aday, 2018). Taken together, this research strengthens the notion that social significance is an important factor in guiding attention in social scenarios, acting as a sort of a semantic “anchor” to elicit selective responses in complex social environments (Capozzi & Ristic, 2018; see also Capozzi et al., 2019). An interesting question for future research is whether similar effects also occur in other contexts, such as non-social domains, in which various forms of information relevance may act as attentional anchors in the presence of crowded and/or inconsistent cues (e.g., Wolfe & Horowitz, 2017). One could test this notion by examining how participants select and respond to conflicting symbolic cues, such as arrows or road signs both in complex real-world scenarios such as in navigating a novel city street as well as in laboratory tests that similarly manipulate task relevance of similar symbols. Thus, future investigations of how learning different forms of social and behavioural relevance (e.g., facial emotions vs. reliability; Dalmaso et al., 2020) may modulate attentional responses and behaviour in a variety of contexts presenting multiple forms of inconsistent cues (e.g., social vs. non-social; Ristic et al., 2012) bode well for understanding how attentional selection occurs in complex situations.

Thus, overall, our study shows that previously learn-ed social information informs subsequent attentional responses in complex multi-agent settings. The implications of these findings dovetail with recent models of social perception that emphasise the downstream consequences of fast social categorizations processes based on minimal social interactions (e.g., Freeman & Johnson, 2016). They also extend these models by suggesting that the consequences of such rapid social categorization extend to attentional orienting processes in a selective and dynamic way by establishing the relevance of individual social cues in multi-agent social settings (see also Capozzi et al., 2018, 2021). In this respect, whereas in the present study we only utilised male face stimuli, which helped us to establish the existence of these effects in a controlled scenario, future research will benefit from investigations of how the characteristics of participants along with the characteristics of stimuli (e.g., including but not limited to gender, see Ciardo et al., 2014) may modulate the interactions between cue perception, social learning, and attention.

In sum, this study shows that both social cue numerosity and previously learned social information guide attention in multi-agent contexts. Thus, whereas sometimes the quantity of social information wins, the perceived quality of individual social information is able to offset the cue numerosity’s powerful effects.

## Supplemental Material

sj-pdf-1-qjp-10.1177_17470218211013028 – Supplemental material for Standing out from the crowd: Both cue numerosity and social information affect attention in multi-agent contexts Supplemental material, sj-pdf-1-qjp-10.1177_17470218211013028 for Standing out from the crowd: Both cue numerosity and social information affect attention in multi-agent contexts by Francesca Capozzi, Andrew P Bayliss and Jelena Ristic in Quarterly Journal of Experimental Psychology

## Notes

1. Following from past work, after the learning phase we asked participants to rate each face image on attributes of friendliness, trustworthiness, attractiveness, dominance, familiarity, and leadership. Participants rated each face on each attribute using a 1 (low) to 9 (high) Likert-type scale. The faces were presented randomly one at a time with the rating question appearing on the top of the screen (e.g., “How *friendly* does this person look in your opinion?”) and the rating scale at the bottom of the screen. The ANOVAs comparing informative-valid, informative-invalid, and uninformative identities yielded no significant effects for the two attributes of most interest friendliness, *F*(2, 148) = .984, *p* = .376, $\eta_{p}^{2}$ = .013, and trustworthiness, *F*(2, 148) = 2.018, *p* = .137, $\eta_{p}^{2}$ = .027, with the other attributes showing similar effects (.355 < *F*s < 2.041, .134 < *p*s < .702). Average ratings are shown in Supplemental Material.
