# Supplementary material for: Standing out from the crowd: Both cue numerosity and social information affect attention in multi-agent contexts
Source: Q J Exp Psychol (Hove). 2021 Apr 29;74(10):1737–46. doi: 10.1177/17470218211013028 (PMC8392755; doi:10.1177/17470218211013028)
Supplement: sj-pdf-1-qjp-10.1177_17470218211013028 – Supplemental material for Standing out from the crowd: Both cue numerosity and social information affect attention in multi-agent contexts [file sj-pdf-1-qjp-10.1177_17470218211013028.pdf]

Standing out from the crowd:

Both cue numerosity and social information affect attention in multi-agent contexts

Francesca Capozzi [a*], Andrew P. Bayliss [b], & Jelena Ristic [a]

a Department of Psychology, McGill University, 1205 Dr Penfield Avenue, Montreal, QC, Canada, H3A 1B1

b School of Psychology, University of East Anglia, Norwich Research Park, Norwich, Norfolk, UK, NR4 7TJ

* corresponding author:

email: francesca.capozzi@mail.mcgill.ca

address: Department of Psychology, McGill University, 1205 Dr Penfield Avenue, Montreal, QC, Canada, H3A 1B1

phone: +1 514 398 1079

SUPPLEMENTARY MATERIAL

Table 1. Average ratings of the stimulus faces

Social attribute	Type of social information		
	Informative-valid identity	Informative-invalid identity	Uninformative identities[a]
Friendliness	4.25 [3.83, 4.68]	4.16 [3.73, 4.59]	4.49 [4.24, 4.74]
Trustworthiness	3.85 [3.52, 4.19]	4.05 [3.66, 4.44]	4.28 [4.05, 4.50]
Attractiveness	3.61 [3.15, 4.08]	3.79 [3.30, 4.48]	3.48 [3.19, 3.76]
Dominance	4.57 [4.08, 5.07]	5.12 [4.66, 5.58]	4.80 [4.52, 5.09]
Familiarity	4.96 [4.38, 5.54]	5.04 [4.46, 5.62]	5.20 [4.74, 5.67]
Leadership	4.51 [4.11, 4.91]	4.71 [4.32, 5.09]	4.59 [4.31, 4.88]

Note. Values in [brackets] are 95% confidence intervals.

[a]Averaged across the three uninformative identities
